# The Two-Component Locus MSMEG_0244/0246 Together With MSMEG_0243 Affects Biofilm Assembly in *M. smegmatis* Correlating With Changes in Phosphatidylinositol Mannosides Acylation

**DOI:** 10.3389/fmicb.2020.570606

**Published:** 2020-09-11

**Authors:** Miaomaio Li, Henrich Gašparovič, Xing Weng, Si Chen, Jana Korduláková, Claudia Jessen-Trefzer

**Affiliations:** ^1^Department of Pharmaceutical Biology and Biotechnology, University of Freiburg, Freiburg, Germany; ^2^Department of Biochemistry, Faculty of Natural Sciences, Comenius University in Bratislava, Bratislava, Slovakia

**Keywords:** mycobacterium, biofilm, two component system, phosphatidylinositol mannosides, membrane lipid, lipid homeostasis, acylation, heme binding

## Abstract

Ferric and ferrous iron is an essential transition metal for growth of many bacterial species including mycobacteria. The genomic region *msmeg_0234* to *msmeg_0252* from *Mycobacterium smegmatis* is putatively involved in iron/heme metabolism. We investigate the genes encoding the presumed two component system MSMEG_0244/MSMEG_0246, the neighboring gene *msmeg_0243* and their involvement in this process. We show that purified MSMEG_0243 indeed is a heme binding protein. Deletion of *msmeg_0243*/*msmeg_0244/msmeg_0246* in *Mycobacterium smegmatis* leads to a defect in biofilm formation and colony growth on solid agar, however, this phenotype is independent of the supplied iron source. Further, analysis of the corresponding mutant and its lipids reveals that changes in morphology and biofilm formation correlate with altered acylation patterns of phosphatidylinositol mannosides (PIMs). We provide the first evidence that *msmeg_0244/msmeg_0246* work in concert in cellular lipid homeostasis, especially in the maintenance of PIMs, with the heme-binding protein MSMEG_0243 as potential partner.

## Introduction

Fatty acids and derived lipid species are essential components of the mycobacterial cell envelope (Marrakchi et al., 2014; Daffé and Marrakchi, 2019). Several genes involved in the metabolism of lipids were shown to be essential in mycobacteria and alterations in lipid compositions are affecting viability and virulence of *tubercle bacilli* (*Mycobacterium tuberculosis, M. tuberculosis, M.tb*), the causative agent of tuberculosis (TB). Two intensively studied proteins in lipid metabolism are the close homologs membrane proteins MmpL11 (Domenech et al., 2005; Pacheco et al., 2013; Wright et al., 2017) and MmpL3 (Grzegorzewicz et al., 2012; Tahlan et al., 2012; Varela et al., 2012; Belardinelli et al., 2016, 2019; Degiacomi et al., 2017; McNeil et al., 2017; Bothra et al., 2018), which are described as part of a lipid export machinery. Furthermore, MmpL3 has been shown to be a target of the pre-clinical candidate drug SQ109 (Tahlan et al., 2012) and other, structurally distinct molecules (Grzegorzewicz et al., 2012). Yet, several reports describe MmpL3 and MmpL11 as heme uptake transporter. In 2011, Tullius et al. (2011) were able to identify a mycobacterial heme import system from *M.tb*. In their studies, several proteins were implicated in playing a role in heme uptake, namely MmpL3 (Rv0206c), MmpL11 (Rv0202c) and the periplasmic protein Rv0203 (Tullius et al., 2011). Rv0203 was identified as extracellular protein that binds heme with a K_d_ of 5.4 × 10^–6^ M. It has a unique structural fold and is proposed to function as a substrate binding protein, grabbing heme in the extracellular space and guiding the molecule to MmpL3/11 for subsequent uptake (Owens et al., 2013, 2012). Recently, Pal et al. (2018) reported abrogated MmpL3 driven mycolic acid transport under iron ion deprivation providing a first link between the dual assigned roles of MmpL3 as metal importer and lipid exporter. Recent studies of Mitra et al. (2017, 2019) implied that a second set of proteins (Ppe36, Ppe62, FecB2, and DppA) is potentially involved in heme import mechanisms in mycobacteria.

The homologs of MmpL3 and MmpL11 in the fast growing non-pathogenic model organism *Mycobacterium smegmatis* (*M. smegmatis, M.sm*) are MSMEG_0250 and MSMEG_0241 (termed MmpL3_S__M_/MmpL11_S__M_ in the following), respectively (McNeil et al., 2017). Interestingly, when comparing the genetic locus surrounding *mmpL3* and *mmpL11* in *M.tb* and *M.sm* genomes, an additional five genes (*msmeg_0243*-*msmeg_0247*) are present only in the *M.sm* genome. The corresponding gene products are a putative heme-binding protein (MSMEG_0243; GenBank accession: ABK72092.1 = “Haemophore, haem-binding; pfam16525”), a two component system (MSMEG_0244/MSMEG_0246), a transmembrane protein (MSMEG_0245) and a secreted peptidase (MSMEG_0247). Upon analyzing mycobacterial genomes using Kyoto Encyclopedia of Genes and Genomes (KEGG, Supporting Dataset 1), we noticed that several other fast-growing species from the phylum such as *Mycobacterium goodie*, *Mycobacterium fortuitum*, or *Mycobacterium phlei* possess homologs of these genes within the heme uptake region. Especially the presence of a two component system including a putative extracellular heme-binding protein within the same locus fueled our interest in deeper investigation of these genes.

Two component systems are known to play pivotal roles in signaling mechanism and are major regulatory systems for bacterial adaptation to environmental changes. Classically, theses machineries consist of two proteins: a membrane bound histidine kinase, which, once a stimulus from the outside has been received, channels this information to its cytosolic response receiver that mediates cellular response mostly *via* gene expression (Beier and Gross, 2006). Two component systems are a prevalent mode of heme sensing in Gram-positive bacteria. Two well-studied examples are the HssRS system of *Staphylococcus aureus* (Stauff and Skaar, 2009), as well as the HrrSA/ChrSA systems of corynebacteria (Heyer et al., 2012; Burgos and Schmitt, 2016), the latter of which appears to play a global role in the control on heme homeostasis. Hence, reading the adjacent gene context MSMEG_0244/MSMEG_0246 might be involved in heme/iron ion sensing or lipid homeostasis. More importantly, a number of Actinobacteria possess unusual two component systems, which depend on (heme-binding) accessory proteins, e.g., HbpS/SenS/SenR in *Streptomyces reticuli* (Ortiz de Orué Lucana and Groves, 2009), or CseA/CseB/CseC in *Streptomyces coelicolor* (Hutchings et al., 2006) with some of these being involved in iron or redox sensing. Furthermore, such systems have been identified in *Bacillus subtilis*, e.g., the essential two component system YycFG that is regulated by YycH and YycI (Szurmant et al., 2007). The accessory proteins are usually lipoproteins, membrane anchored or periplasmic proteins found in the same operon.

MSMEG_0244 and MSMEG_0246 are annotated as transcriptional regulatory protein PrrA and sensor-type histidine kinase PrrB. PrrA/PrrB (RegA/RegB) with the accessory protein PrrC is a well-studied global regulatory system in *Rhodobacter sphaeroides* involved in the regulation of photosynthesis genes, CO_2_ and nitrogen fixation, denitrification, negative aerotactic response, electron transfer, and aerobic respiration (McEwan et al., 2002). It is noteworthy that PrrA/PrrB is proposed to receive an inhibitory signal from the electron transport chain mediated by PrrC, a copper-binding protein, and this signal transduction process may involve oxidation−reduction reactions (McEwan et al., 2002). The closest homolog of MSMEG_0244/MSMEG_0246 in *M.tb* is Rv0903c/Rv0902c (PrrA/PrrB), a two component system shown to be essential for intracellular growth of *tubercle bacilli* (Ewann et al., 2002, 2004). It was suggested that PrrA/PrrB may play a role in regulating gene expression in *M.tb* during nitrogen-limiting conditions (Haydel et al., 2012), however, possible signaling molecules for PrrA/PrrB have not been reported and a crystal structure of full-length PrrA (Pdb code: 1YS6) and a truncated version of PrrB (Pdb code: 1YSR) are only available as apo-proteins (Nowak et al., 2006). PrrA/PrrB has been proposed as highly attractive drug target (Bellale et al., 2014).

*M.sm* possesses a paralog of MSMEG_0244/MSMEG_0246, encoded by *msmeg_5662/msmeg_5663* genes, which is a closer homolog of PrrA_tb_/PrrB_tb_. MSMEG_5662/MSMEG_5663 was initially described as being essential in *M.sm* (Mishra et al., 2017), however, according to a more recent publication the locus *msmeg_5662/msmeg_5663* was successfully deleted in *M.sm*, establishing that the PrrA/PrrB system is not universally essential. The mutant strain exhibited clumping in ammonium-limited medium and significantly reduced growth during ammonium and hypoxic stress and was shown to influence triacylglycerol species during ammonium stress in *M.sm* (Maarsingh and Haydel, 2018). Transcriptome analysis of a *msmeg_5662/msmeg_5663* knockout mutant provided first evidence that the two-component system regulates respiratory and oxidative phosphorylation pathways and positively regulates expression of the dormancy-associated DosR response regulator genes in an oxygen independent manner (Maarsingh et al., 2019).

In this study, we have delineated the above described genomic region including the genes encoding dual histidine kinase *msmeg_0244/msmeg_0246* and the adjacent gene *msmeg_0243*, and its potential role in heme/iron sensing. Firstly, we verified heme binding of purified MSMEG_0243. Then we constructed three knockout mutants Δ*msmeg_0243*, Δ*msmeg_0244/msmeg_0246* and Δ*msmeg_0243/msmeg_0244/msmeg_0246*. We analyzed bacterial growth of the knockout mutants in the presence of different iron (III) sources. We found that the knockout mutant Δ*msmeg_0243/msmeg_0244/msmeg_0246* shows reduced biofilm formation and abrogated colony morphology independent of the supplied iron source. Further investigations on the lipid composition of this mutant identified an altered acylation-state of cell envelope phosphatidylinositol mannosides (PIMs). Our data provide insights into a putative role of MSMEG_0244/MSMEG_0246 and the potential accessory and heme-binding protein MSMEG_0243 in regulation of PIMs acylation.

## Experimental Procedures

### Strains, Plasmids, and Growth Conditions

Strains, plasmids and primers used in this study are listed in Supplementary Table S1. Routinely, *M. smegmatis* mc^2^155 or derived strains were cultured in 7H9 broth (Sigma-Aldrich; ingredients: 0.5 g/L ammonium sulfate, 2.5 g/L disodium phosphate, 1.0 g/L monopotassium phosphate, 0.1 g/L sodium citrate, 0.05 g/L magnesium sulfate, 0.5 mg/L calcium chloride, 1 mg/L zinc sulfate, 1 mg/L copper sulfate, 0.04 g/L ferric ammonium citrate, 0.05 g/L L-glutamic acid, 1 mg/L pyridoxine, 0.5 mg/L biotin) supplemented with 10% (v/v) ADC (2% dextrose, 5% albumin, 0.85% NaCl) and 0.05% (v/v) Tween 80. To obtain growth curves in the absence of iron or at low iron (FeCl_3_ or hemin) concentrations, cells were pre-cultured in 7H9 medium (lacking ADC) and subsequently transferred into Fe(III)-free modified Sauton’s medium (0.5 g/L monobasic potassium phosphate, 0.5 g/L magnesium sulfate, 4.0 g/L L-asparagine, 2.0 g/L citric acid, 47.6 mL/L glycerol, and 0.1 mL/L 1% zinc sulfate solution, pH 7.2). Cells were sub-cultured five times to deplete intracellular iron pools (Mitra et al., 2019). For cloning procedures, *Escherichia coli* Xl1-Blue was grown in Luria Bertani (LB) broth and LB-agar. For selection purposes kanamycin and/or hygromycin B were used at final concentrations of 50 μg/mL.

*Mycobacterium smegmatis* mc^2^155 has been handled in a Biosafety 2 Laboratory in accordance to German Federal Law.

### Predictions and BLAST Alignment

Homologs/Paralogs were identified using blastp suite (National Library of Medicine). Secretion signal peptide predictions were performed using SignalP 5.0 Server (Technical University of Denmark). Operon analysis was performed using OperonDB, Door^2^ and Microbes online.

#### Cloning of pET-22(b) + -^Strep^MSMEG_0243

The open reading frame encoding truncated MSMEG_0243 was amplified from genomic DNA of *M.sm* mc^2^155 using the primer pair MSMEG_0243_fw/rev as indicated in Supplementary Table S1. The obtained PCR product and pET-22(b) + were digested with *Nco*I and *Hin*dIII and ligated using T4 DNA ligase. After transformation, colonies were selected on LB- agar plates with Ampicillin and sequenced to validate correct sequence incorporation.

### Purification of ^Strep^MSMEG_0243

Plasmid pET-28(b) + -^Strep^MSMEG_0243 was transformed into chemically competent *E. coli* Bl21 Rosetta cells and transformants were selected on LB plates containing Ampicillin. Single colonies were isolated and validated by PCR analysis. One colony was transferred into LB-medium (containing Ampicillin) and grown over night at 37°C. One milliliter pre-culture was transferred into 200 mL auto-inducing medium (10 g/L N-Z amine AS, 5 g/L yeast extract, 0.5 g/L glycerol, 50 mg/L glucose, 0.2 g/L lactose, 0.33 g/L (NH_4_)SO_4_, 0.68 g/L KH_2_PO_4_, 0.71 g/L Na_2_HPO_4_, 1 mL of 1 M MgSO_4_, 1 mL of 1000x trace elements mix) and grown for 2 days at 28°C. Cells were harvested by centrifugation and cells were lysed in cell lysis buffer (50 mM NaH_2_PO_4_, 300 mM NaCl, 1 mM DTT, and 1 mM PMSF, 1 mg/mL lysozyme, pH 8.0) by sonication. The obtained crude cell lysate was centrifuged and the supernatant was applied on Streptavidin beads (250 μL 50% slurry/mL lysate). Subsequently, Streptavidin beads were washed with wash buffer (20 mM NaH_2_PO_4_, 100 mM NaCl, 0.05% glycerol, pH 7.4) to remove any non-specifically bound proteins. Proteins were eluted from the beads using elution buffer (20 mM NaH_2_PO_4_, 100 mM NaCl, 0.05% glycerol, 2.5 mM desthiobiotin, pH 7.4). The obtained fractions were analyzed by SDS-PAGE, pooled, concentrated and dialyzed against 20 mM Tris-HCl, 100 mM NaCl, 0.05% glycerol, pH 7.4 overnight at 4°C. Further purification was achieved by size exclusion chromatography on a HiLoad Superdex 200 PG column (Äkta Protein Purification System, GE Healthcare Germany). Protein was eluted from the column in 20 mM Tris-HCl, 100 mM NaCl, pH 7.4. Fractions were analyzed by SDS-PAGE, pooled, concentrated and stored at −80°C until further use.

### Heme Titration Experiments

Concentrated purified protein ^Strep^MSMEG_0243 (3 mg/mL) was transferred into a hypoxic chamber and diluted in assay buffer (20 mM Tris-HCl, 100 mM NaCl, 0.05% glycerol, pH 7.4) in a cuvette. Spectra were recorded on a JASCO V-650 Spectrophotometer (JASCO, Germany). Subsequently, increasing equivalents of hemin (reconstituted freshly in DMSO under oxygen limiting conditions at 10 mM concentration and further diluted in assay buffer) were added and incubated for 5 min, until, after recording of the spectra, an additional equivalent of hemin was introduced. Samples were reduced by the addition of 0.2 mM dithionite. Heme:protein ratio was determined by recording spectra for hemin only at the indicated concentrations and apo-protein (10 μM), which was subsequently saturated with increasing hemin concentrations. Delta absorbance values at 420 nm were calculated using Spectra Manager^TM^ II Software and Excel 2016.

### *M. smegmatis* Mutant Construction

Knockout mutants were constructed using the pGOAL19/p1NIL system as published previously by Parish and Stoker (2000). Upstream and downstream fragments of the genomic loci were amplified from genomic DNA of *M. smegmatis* mc^2^155 using the primer pairs as indicated in Supplementary Table S1. The obtained fragments were cloned into *Pac*I and *Kpn*I restrictions sites of p1NIL for the upstream fragment and *Kpn*I and *Hin*dIII restrictions sites for the downstream fragment. Transformants were selected on LB plates containing kanamycin, screened by PCR and further validated by Sanger sequencing. Subsequently, the resistance marker cassette from pGOAL19 was cloned into the *Pac*I site of p1NIL-Δ0243 (or Δ0244/0246 or Δ0243/0244/0246) to give the final suicide vector p1NIL-Δ0243 (or Δ0244/0246 or Δ0243/0244/0246)-RM. Vector DNA was pretreated with 100 mJ UV light cm^–2^ and used to transform electro-competent *M.sm* mc^2^155 cells. Single cross-over events were selected on 7H9 agar containing kanamycin and hygromycin B. Single colonies were re-streaked on X-GAL plates and blue colonies were isolated and analyzed by PCR. One representative single cross-over mutant was selected and incubated in liquid 7H9 medium containing 10% sucrose and plated in serial dilutions on 7H9 X-GAL plates to screen for second-crossover events. White colonies were analyzed by PCR to distinguish between *wild-type* and knockout clones. The obtained double cross-over mutant was further validated by qRT-PCR analysis using the primer pair as indicated in Supplementary Table S1.

### Complementation of *M. smegmatis* Δ0243/0244/0246

The *msmeg_0243-0246* locus comprising an additional 800-bp upstream fragment containing the putative native promoter region was amplified from genomic DNA by PCR using the oligonucleotide pair as indicated in Supplementary Table S1 and cloned into the single-copy integrative plasmid pJAK1.A (Kan) *via* Gateway Technology (Invitrogen), following the instructions by the manufacturer. The plasmid was transformed into the mutant by electroporation. Transformants were selected on 7H9 agar plates containing kanamycin, screened by PCR and further validated by Sanger sequencing.

### Growth Curve Analysis and Pellet Dry Weight Assessment

Iron depleted cells were diluted into modified Sauton’s medium at a starting OD_600_ of 0.01. Medium was supplemented with either 1 μM FeCl_3_ or 1 μM hemin. Recording of OD_750_ at the indicated time points was used to monitor growth. To disperse cells, suspensions were passed through a syringe (26-gauge needle) several times prior to the measurements. In order to determine the dry weight of cells pellets, iron depleted cells were diluted into modified Sauton’s medium at a starting OD_600_ of 0.01. Medium was supplemented with 1 μM hemin. After 65 h, cells were spun down and the supernatant was discarded. Cell pellets were subsequently dried in an oven at 65°C for 1 day and pellet weight was determined using an analytical balance.

### Minimal Inhibitory Concentration (MIC)

MIC was determined using the resazurin reduction method as described elsewhere (Taneja and Tyagi, 2007). Briefly, cells were grown in the indicated medium and harvested at mid-log OD_600_. Cells were diluted to OD_600_ 0.05 and incubated with different concentrations of inhibitor in a 96-well plate in a final volume of 200 μL. After 24 h, 20 μL resazurin (0.15 mg/mL) were added and color change was observed after 24 h (ex/em: 560 nm/590 nm). As a positive control ciprofloxacin was used. Fluorescence relative units were converted into % viability by normalizing to control well reads containing cells only and DMSO. OriginLab Origin 2019b software was used to generate viability curves and fitting of curves to determine IC50 values was achieved by applying the sigmoidal fitting function “DoseResp.”

### Biofilm Growth

Cultures were diluted into modified Sauton’s medium containing 10 μM FeCl_3_ or 10 μM hemin or no supplement at a starting OD_600_ of 0.006. In the absence of Tween 80, standing cultures were grown in 12-well dishes to promote biofilm formation in a humidified incubator at 30°C for 4 days. For lipid analysis the cultures were diluted into standard Sauton’s medium or M63 salts (Recht and Kolter, 2001) at a starting OD_600_ of 0.001 and grew 3 days at 37°C (Sauton) or 5 days at 30°C (M63).

### Growth on Solid Media

Cultures were diluted into 7H9 liquid medium without Tween 80 supplement at a starting OD_600_ of 0.1, 0.01 and 0.001. 1 μL starting culture was pipetted in the center of the plate on 7H9 agar (no Tween 80) and grown in an incubator at 37°C for 6 days. For lipid analysis the cultures were diluted to OD_600_ of 0.01, 0.001 and 0.0001 and 4 μL of each culture was pipetted on 7H9 agar supplemented with 10% (v/v) ADC or 7H11 agar supplemented with 10% Middlebrook oleic acid-albumin-dextrose-catalase (OADC) enrichment. The plates were cultivated 3 days at 37°C. For the cultures carrying pJAK1.A vector kanamycin was used at final concentration of 20 μg/mL.

### RNA Extraction

Cultures were grown to mid-log phase in 7H9 medium. Cells were collected by centrifugation. Cell pellets were flash-frozen in liquid nitrogen and stored at −80°C until further use. Total RNA was isolated using the RNeasy Mini-Kit (Qiagen) following the manufacturer’s instructions. Amount and quality of the RNA were assessed spectrophotometrically. Integrity of each RNA sample was assessed by agarose gel electrophoresis. RNA was stored at −80°C in nuclease free water.

### Quantitative Reverse Transcription-PCR (qRT-PCR)

qRT-PCR analysis was performed with the primer pairs described in Supplementary Table S1, using the SYBR^®^ Green PCR Master Mix (Thermo Fisher Scientific) according to the protocol provided by the supplier. SigA primers were used as a control.

### Operon Analysis by RT-PCR

RT-PCR analysis was performed with the primer pairs described in Supplementary Table S1. cDNA was synthesized using the ProtoScript^®^ first strand cDNA synthesis kit (Qiagen, Germany) according to the protocol provided by the manufacturer. SigA primers were used as a control. Control reactions were performed in the absence of reverse transcriptase. Subsequently, 1 μg of cDNA was used in each reaction, supplemented with the Phusion DNA Polymerase, reaction buffer and intergenic region specific primers (see Supplementary Table S1). A standard PCR protocol was used in subsequent reactions: initial denaturation (95°C, 2 min) followed by 35 cycles of denaturation (95°C, 15 s), annealing (60°C, 30 s) and extension (72°C, 15 s) with a final extension step (72°C, 5 min).

### Lipid and Mycolic Acids Analysis by Thin-Layer Chromatography (TLC)

The cell pellets were resuspended in 6 mL of chloroform/methanol (1:2) and extracted for 2 h at 56°C with shaking. After centrifugation at 2200 × g, the supernatants were stored and subsequently the pellets were extracted twice with chloroform/methanol (2:1), each extraction at 56°C for 2 h. Supernatants of all three extractions were pooled, dried under the stream of nitrogen and washed in chloroform/methanol/water (4/2/1) as described by Folch (Folch et al., 1957). The organic layers were collected, dried under nitrogen and lipids were dissolved in chloroform/methanol (2:1) – 600 μL per 0.5 g of wet cell pellets. Five microliter were loaded on thin-layer chromatography (TLC) silica gel plates F254 (Merck) and the lipids were separated in solvent I (chloroform: methanol: ammonium hydroxide: water; [65:25:0.5:4]; solvent II (chloroform: methanol: water [20:4:0.5] and solvent III (petroleum ether: ethylacetate [95:5; 3 runs]). Lipids were visualized with CuSO_4_ (10% CuSO_4_ in 8% phosphoric acid solution) or 0.5% (w/v) α-naphtol in 5% (v/v) sulphuric acid in ethanol and heating. The intensity of the spots on the TLC was quantified using ImageJ software (Schneider et al., 2012).

Methyl esters of fatty acids (FAME) and mycolic acids (MAME) were prepared as previously described (Phetsuksiri et al., 1999). Samples were dissolved in chloroform:methanol (2:1) and loaded on TLC plates as described for lipid extracts. Different forms of methyl esters were separated in solvent IV (*n*-hexane: ethyl acetate [95:5; 3 runs]) and detected as described above.

## Results

### Gene Cluster Analysis and Protein Alignment

**FIGURE 1:**
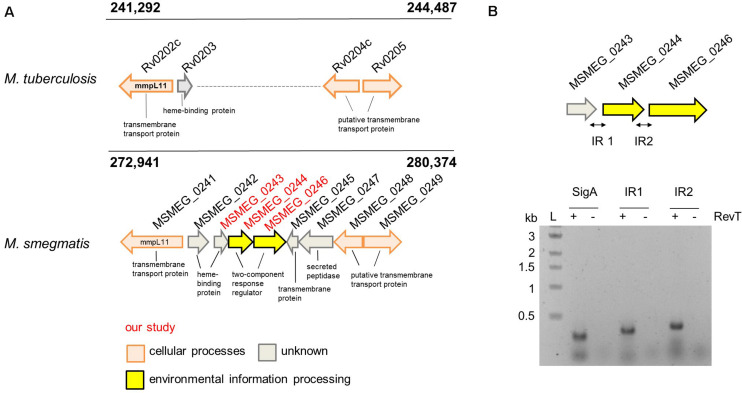
The potential heme uptake and lipid export locus in *M. tuberculosis* and *M. smegmatis*. **(A)** Alignment of the genomic region surrounding additional five genes located on the *M. sm* genome. Highlighted in red: the three genes investigated in our study. **(B)** Operon analysis by RT-PCR of *msmeg_0243*, *msmeg_0244*, and *msmeg_0246* confirmed the co-transcription of these three genes. Analysis was performed in the presence (+) or absence (−) of reverse transcriptase (RevT). IR, intergenic region. Expected PCR product size IR1: 336 bp, IR2: 378 bp.

KEGG analysis using the predicted gene cluster of the putative “heme-uptake region” surrounding *mmpL3* and *mmpL11* genes in *M.tb* as input sequence identified the presence of orthologs regions throughout the mycobacterium *genus* (Supplementary Data Sheet 2). Close analysis of the corresponding alignment revealed in several mycobacterial species an additional genetic stretch consisting of five genes located between *mmpL3* and *mmpL11*, e.g., in *M.sm*, a putative heme-binding protein (*msmeg_0243*), a two component system (*msmeg_0244/msmeg_0246*), a transmembrane protein (*msmeg_0245*) and a secreted peptidase (*msmeg_0247*) (Figure 1 and Supplementary Figure S1). Operon analysis using three different online databases (OperonDB, Door2 and Microbes) predicted that the two component system *msmeg_0244/msmeg_0246* is transcribed from one operon together with *msmeg_0243* and we subsequently confirmed the *in silico* prediction by RT-PCR (Figure 1). MSMEG_0243 is annotated as periplasmic heme binding protein carrying a signal peptide for translocation *via* the Sec pathway (Natale et al., 2008). The protein is a close homolog of the extracellular heme-binding protein Rv0203 from *M.tb* (42% sequence similarity) and has six paralogs in *M.sm*. Interestingly, one paralog MSMEG_0242, is located directly upstream of MSMEG_0243, and is a heme-binding protein potentially located in the periplasm. KEGG analysis determined one paralog of MSMEG_0244/MSMEG_0246 in *M.sm* (MSMEG_5662/MSMEG_5663) and homologs throughout the mycobacterium *genus* including *M.tb*. Pairwise alignment between the *M.sm* and *M.tb* proteins revealed a 54% similarity between PrrB_tb_ (Rv0902c) and MSMEG_0246 and a 70% similarity between PrrA_tb_ (Rv0903c) and MSMEG_0244.

### Purification and Characterization of MSMEG_0243

In order to investigate the function of the two component system MSMEG_0244/MSMEG_0246 and its potential accessory protein MSMEG_0243, at first, we decided to validate the predicted heme binding of MSMEG_0243 *in vitro*.

**FIGURE 2:**
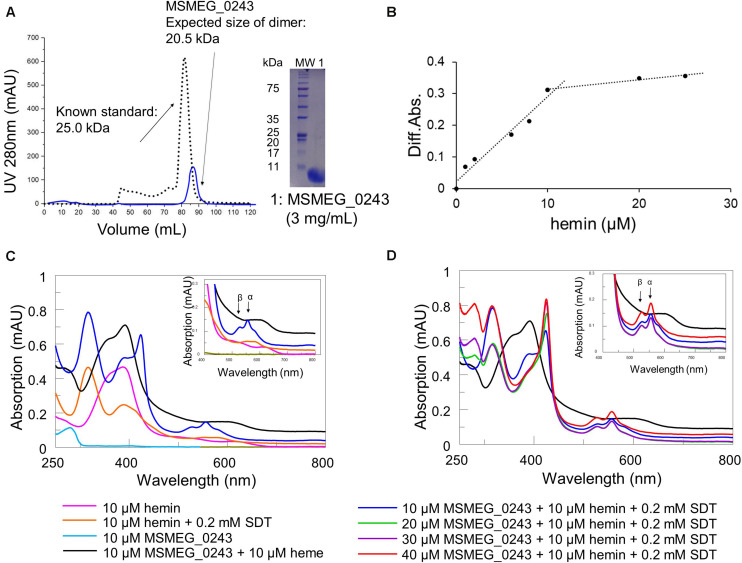
Purification and heme binding of MSMEG_0243. **(A)** Purification of MSMEG_0243 in its apo form. Size exclusion chromatography was performed after purification of MSMEG_0243 *via* Strep-tag. The elution profile (blue line) and subsequent SDS-PAGE analysis of the combined elution fractions are shown. Overlay of the chromatogram of MSMEG_0243 (blue line, size of dimer: 20.5 kDa) with a profile from a protein with known size (black dotted line, 25.0 kDa) verified the expected size for a protein dimer. **(B)** Titration of hemin to apo- MSMEG_0243 (10 μM) is observed by the spectral change at 420 nm between protein plus hemin and hemin only. The protein saturates at 10 μM hemin, indicating a 1:1 binding ratio. **(C)** Absorbance spectrum of apo- MSMEG_0243 (light blue line) and heme-bound MSMEG_0243 (black line) and comparison to hemin only (pink line). Reduction by the addition of 0.2 mM sodium dithionite (dark blue line) shifts the Soret band from 392 to 424 nm. Control conditions are hemin only plus 0.2 mM sodium dithionite (orange line). **(D)** Increasing concentrations of apo- MSMEG_0243 were added to the reduced heme- MSMEG_0243 complex. Represented by the blue line that shifts to (i) light green (20 μM), (ii) violet (30 μM), and (iii) red line (40 μM). The observed peak increase at 392 nm potentially is a result of ferrous heme dissociating from MSMEG_0243. All spectra were recorded under oxygen free conditions in a hypoxic chamber. The insets show the magnified Q-band region. All experiments were performed at least three times and one representative dataset is shown. SDT: sodium dithionite.

We cloned and expressed the corresponding gene and purified the protein to near homogeneity from *Escherichia coli* (*E. coli*) cell lysates. Purification was achieved *via* Strep-tag of a truncated version of MSMEG_0243 lacking the predicted signal peptide (cleavage site between pos. 25 and 26: ALA-DP; probability: 0.9537), followed by size exclusion column chromatography (Figure 2A), revealing isolation of a protein dimer. Analysis of the purified protein *via* UV/Vis spectroscopy revealed the absence of a characteristic heme absorbance maximum at 410 nm, indicating purification of the apo protein (Figure 2C). However, titration experiments with chloroprotoporphyrin IX iron(III) (Fe-PIX, hemin), a well-established heme substitute (Tullius et al., 2011), and subsequent UV/Vis absorption spectroscopy validated heme binding of MSMEG_0243 (Figure 2C). Initially, we titrated hemin to a fixed concentration of protein in order to determine binding stoichiometry and subsequently, we calculated a heme:protein ratio of 1:1 (Figure 2B). Under oxidizing conditions the protein-heme complex showed a Soret maximum at 392 nm with a shoulder at 360 nm and a broad peak at 600 nm (Figure 2C). The obtained spectrum with a Soret band below 400 nm and a charge transfer band near 600 nm indicates that heme iron is high spin (*S* = 5/2). Anaerobic reduction by addition of dithionite produces a characteristic red-shifted Soret peak. The observed peak is broad with two maxima at 388 and 424 nm, potentially indicating that heme has partially dissociated from the protein, as it has been observed in similar experiments for Rv0203, the MSMEG_0243 homolog from *M.tb*. Upon subsequent titration of 3 equivalents apo-MSMEG_0243, the peak at 424 nm reaches a maximum, while the shoulder at 388 nm becomes less apparent (Figure 2D). Most likely, as proposed for Rv0203, MSMEG_0243 binds less tight to the ferrous as to the ferric heme molecules, which leads to increased dissociation rates. Under reducing conditions, only one distinct α-band (557 nm) and a shoulder in the β-band region (527 nm) was observed.

Overall, our results are in line with published data on Rv0203, indicating heme binding to both proteins in a comparable fashion (Tullius et al., 2011).

### Generation and Growth Characteristics of the Knockout Mutants *M. smegmatis* mc^2^155 *Δmsmeg_0243/msmeg_0244/msmeg_0246, Δmsmeg_0244/msmeg _0246*, and *Δmsmeg_0243*

Next, we constructed three knockout mutants to investigate heme dependent phenotypic effects upon loss of *msmeg_0243* or *msmeg_0244/msmeg_0246*. The knockout mutants were constructed in-frame using the pGOAL19/p1NIL system as published previously by Parish and Stoker (2000). Upstream and downstream fragments of the respective genomic locus were amplified from genomic DNA of *M. smegmatis* mc^2^155 using the primer pairs as indicated in Supplementary Table S1. The successful gene deletions were validated by PCR, Sanger Sequencing and RT-PCR analysis (Supplementary Figures S2A,B). In total we constructed three mutants Δ*msmeg_0243*/ *msmeg_0244/msmeg_0246*, Δ*msmeg_0244/msmeg_0246* and Δ*msmeg_0243*.

**FIGURE 3:**
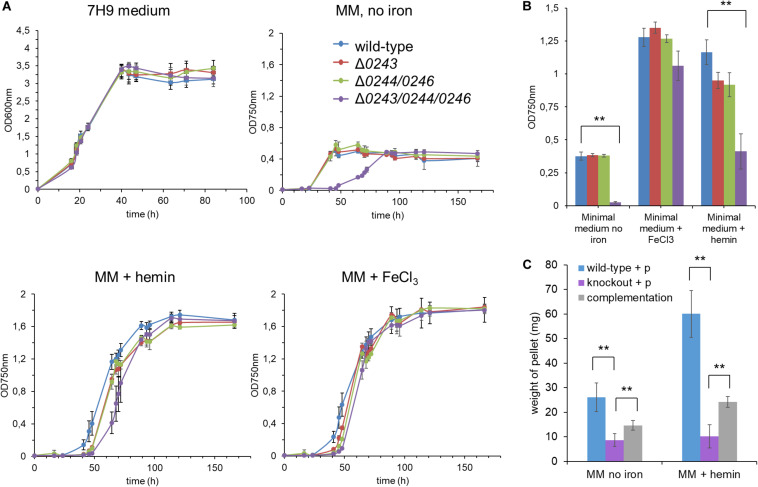
Growth curve analysis of the mutants *M. smegmatis* mc^2^155 Δ*0243/0244/0246*,Δ*0244/0246*, and Δ*0243* compared to wild-type cells. **(A)** Growth was recorded in classical mycobacterial broth “7H9 medium” and optical density was recorded at 600 nm. After 5 rounds of iron starvation, strains were grown in minimal medium (modified Sauton’s medium, MM), either iron free, in 1 μM FeCl_3_ or 1 μM hemin supplement to determine growth dependence on the supplied iron source. Optical density was recorded at 750 nm due to partial hemin absorbance at 600 nm. **(B)** Summary of the growth curve results at the mid-log growth time point in modified Sauton’s medium (@ 45 h for iron free condition and @ 65 h for + iron/+ hemin conditions). Mutant strain Δ*0243/0244/0246* shows significantly reduced growth in hemin as sole iron source and in iron free conditions (ANOVA-Test, **p ≤ 0.05). **(C)** Growth in modified Sauton’s medium + hemin and in iron free conditions was partially restored in the complemented strain. Due to extensive clumping of the complemented strain during iron starvation and subsequent growth in minimal medium supplemented with hemin or no iron source, growth was assessed by determination of dry weight of the resulting pellet. Clumping was not fully resolved by mechanical disruption or related methods. Wild-type + p: *M. smegmatis* mc^2^155:pJAK1.A; knockout + p: *M. smegmatis* mc^2^155 Δ*0243/0244/0246*:pJAKA.1 and complementation: *M. smegmatis* mc^2^155 Δ*0243/0244/0246*:pJAKA.1_*0243/0244/0246*. All experiments were performed in triplicates. Error bar represents ± SEM. Blue: wild-type, red: Δ*0243*, green: Δ*0244/0246*, purple: Δ*0243/0244/0246*, gray: complementation of Δ*0243/0244/0246.*

Upon culturing *M. smegmatis* mc^2^155 wild-type, Δ*msmeg_ 0243*/*msmeg_0244/msmeg_0246, msmeg_0244/msmeg_0246*, or Δ*msmeg_0243* in classical growth medium for mycobacteria (7H9 medium + 0.05% Tween 80), no significant differences in growth could be observed between the strains indicating that the investigated genes are non-essential under the studied conditions (Figures 3A,B). To assess whether the two component system is affected by extracellular iron or heme, subsequent experiments were conducted with iron (III/II)-starved bacteria, which were passaged 5 times in iron (III/II) depleted medium to reduce intracellular iron pools. Culturing the starved bacterial cultures in modified Sauton’s medium without iron resulted in significantly suppressed growth. Addition of either EDTA an iron (III) complexing agent or 2,2′-bipyridine an iron (II) complexing agent did not result in further growth retardation, indicating that no traces of ferric or ferrous ions were influencing bacterial growth. Interestingly, the mutant Δ*msmeg_0243*/*msmeg_0244/msmeg_0246* showed retarded growth under iron limiting conditions as compared to the wild-type strain (Figure 3A). Complementation of the knockout strain did only partially restore growth (Figure 3C), indicating a general fitness loss of the mutant under limiting conditions. Next, we cultured iron (III/II)-starved *M.sm* mc^2^155 wild-type or the mutants Δ*msmeg_0243*/*msmeg_0244/msmeg_0246, msmeg_0244/msmeg_0246*, or Δ*msmeg_0243* in iron (III/II) free medium complemented with iron(III)chloride or protoporphyrin IX iron(III). While in the presence of iron chloride all strains grew to a similar extent, in the presence of hemin we observed a slight but significant growth retardation of Δ*msmeg_0243*/*msmeg_0244/msmeg_0246* during logarithmic growth phase (Figure 3B, *p*-value p ≤ 0.05 at 65 h growth). Complementation of the knockout mutant strain Δ*msmeg_0243*/*msmeg_0244/msmeg_0246* partially restored growth under the applied conditions (Figure 3C).

We thus concluded that minor effects on the growth in the presence of hemin of the investigated mutant are observable in iron starved bacteria and that under classical laboratory conditions these effects are negligible.

Interestingly, Mitra et al. (2017) showed that inactivation of the heme utilization genes *ppe36*, *ppe62*, and *fecB2* in *M.tb* confers resistance to gallium(III)-porphyrin (Ga-PPIX), a toxic heme analog. Hence, we subsequently tested the minimal inhibitory concentration (MIC) of Ga-PPIX in *M.sm* wild-type and our knockout mutants. We rationalized that loss of the putative heme sensing mechanism might affect the sensitivity to this toxic heme substitute. We conducted the assay with non-starved bacteria under three conditions: 7H9 medium (+0.05% Tween 80), modified Sauton’s medium (complemented with 10 μM ironIII + 0.05% Tween 80) and modified Sauton’s medium (iron free + 0.05% Tween 80). Results are summarized in Supplementary Figure S3A. Ciprofloxacin, a fluoroquinolone compound, with high activity against various mycobacteria (Young et al., 1987), was used as a control inhibitor and cell growth was monitored to exclude any interfering growth effect of the described mutants (Supplementary Figure S3B). In none of the conditions tested, we observed a significant difference in MIC when comparing mutants with wild-type cells. These results did not confirm that heme or heme analog sensing/uptake is influenced by MSMEG_0243 or MSMEG_0244/MSMEG_0246, hence these proteins are potentially not involved in the sensing/uptake process or their loss of function might be compensated by homologous proteins in *M.sm*.

### *M. smegmatis* mc^2^155 *Δmsmeg_0243/msmeg_0244/msmeg_0246, Δmsmeg_ 0244/msmeg_0246*, and *Δmsmeg_0243* Are Not Affected by the Presence of Gases or Radicals

Extracellular heme binding proteins, are also known to play various roles in cellular gas or radical sensing, as the iron metal center in heme is able to coordinate and transport O_2_, CO, NO or radical species (Girvan and Munro, 2013; Sivaramakrishnan and Ortiz de Montellano, 2013). In order to rule out a possible involvement of MSMEG_0243/MSMEG_0244/MSMEG_0246 in a gas or radical sensing mechanism we cultured wild-type and mutant strains under standard normoxic laboratory conditions and compared bacterial growth behavior under anoxic, NO or CO releasing conditions and in the presence of H_2_O_2_, a molecule that supports the formation of reactive oxygen species. To that end, we plated serial dilutions of the four strains on 7H9 agar only, or agar containing either the NO donor DETA-NON-Oate, the CO donor CORm-2 or H_2_O_2_. Plates were incubated at 37°C under normoxia or for oxygen limiting conditions in a gas pack chamber. Under none of our conditions tested we could observe a significant difference in growth rate or behavior (Supplementary Figure S4), and thus we concluded that *msmeg_0243/msmeg_0244/msmeg_0246* are, under the applied conditions, not involved in a gas or radical sensing mechanism in mycobacteria.

### Phenotypic Observations Resulting From Growth Analysis of *M. smegmatis* mc^2^ 155 *Δmsmeg_0243/msmeg_0244/msmeg_0246, Δmsmeg_0244/msmeg_ 0246*, and *Δmsmeg_0243*: Biofilm Formation and Colony Formation

**FIGURE 4:**
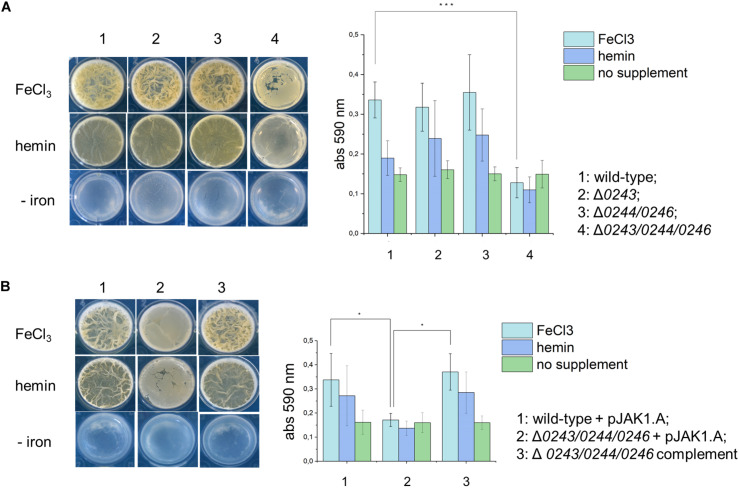
A top-down view of biofilms from *M. smegmatis* mc^2^155 wild-type, knockout mutants and complemented strain in detergent-free Sauton’s medium, either with or without supplemental iron (10 μM FeCl_3_ or 10 μM hemin) after 4 days of incubation. **(A)** 1: wild-type; 2: Δ*0243*; 3: Δ*0244/0246*; 4: Δ*0243/0244/0246*. Bar graph represents biofilm quantification using crystal violet stain from triplicate experiments. Error bars represent ± SEM. ***p< 0.0005 (ANOVA test). **(B)** 1: wild-type + pJAK1.A; 2: Δ*0243/0244/0246* + pJAK1.A; 3: Δ*0243/0244/0246* complemented. Bar graph represents biofilm quantification using crystal violet stain from triplicate experiments. Error bars represent ± SEM. *p < 0.05 (ANOVA test). Light blue: FeCl_3_ supplement, dark blue: hemin supplement and green: no supplement.

Interestingly, we noticed that during culturing *M.sm* wild-type and Δ*msmeg_0243/msmeg_0244/msmeg_0246* in liquid standing cultures, biofilm formation was significantly altered in the knockout strain, as well as colony appearance on solid medium. This prompted us to further study biofilm formation of Δ*msmeg_0243/msmeg_0244/msmeg_0246*, Δ*msmeg_0244/msmeg_0246*, and Δ*msmeg_0243.* We diluted pre-cultures in Sauton‘s medium complemented with either hemin or free iron (III) and incubated the cultures in standing 24-well plates at 30°C in a humidified incubator for 4 days. Biofilm formation was quantified by crystal violet stain (Figure 4A). Both strains, the single mutant Δ*msmeg_0243* and the double mutant *msmeg_0244/msmeg_0246*, did not show any obvious defect in biofilm formation as compared to the wild-type in neither of the conditions tested. To the contrary, the strain Δ*msmeg_0243/msmeg_0244/msmeg_0246* grew significantly less biofilm and formed less structured pellicle when compared to the wild-type strain both in hemin or iron supplemented medium. This effect was especially enhanced at later time points of 4-day growth. The observed effect was restored to wild-type behavior in the complemented mutant strain (Figure 4B). In order to show that the observed effect is not a result of differential bacterial growth, we isolated planktonic grown bacteria at the bottom of the plate, re-suspended them in 7H9 containing Tween 80 and quantified optical density (see Supplementary Figure S5). Clearly, Δ*msmeg_0243/msmeg_0244/msmeg_0246* compensated low biofilm formation by enhanced planktonic growth, while the wild-type strain and the two knockout mutants Δ*msmeg_0243* and *msmeg_0244/msmeg_0246* grew to a similar extend. Our results obtained for Δ*msmeg_0243/msmeg_0244/msmeg_0246* correlate with studies describing the knockout mutant *M.sm*Δ*mmpl11_S__M_*. This strain also showed reduced biofilm formation in standing cultures (Pacheco et al., 2013). In order to investigate any trans-effects of our knockout mutant on *mmpL11_S__M_*, we performed RT-PCR-analysis on Δ*msmeg_0243/msmeg_0244/msmeg_0246* and wild-type cultures, but we did not observe a down-regulation of MmpL11_S__M_ (Supplementary Figure S2C). Instead, RT-PCR analysis revealed upregulation of *mmpL11_S__M_* in all three knockout mutants. Upregulation of *mmpL11* in *M.sm*, however, is linked to fast and robust biofilm formation, further confirming that our observed phenomena of reduced and structurally altered biofilms are not a result of a trans-effect on *mmpl11*.

**FIGURE 5:**
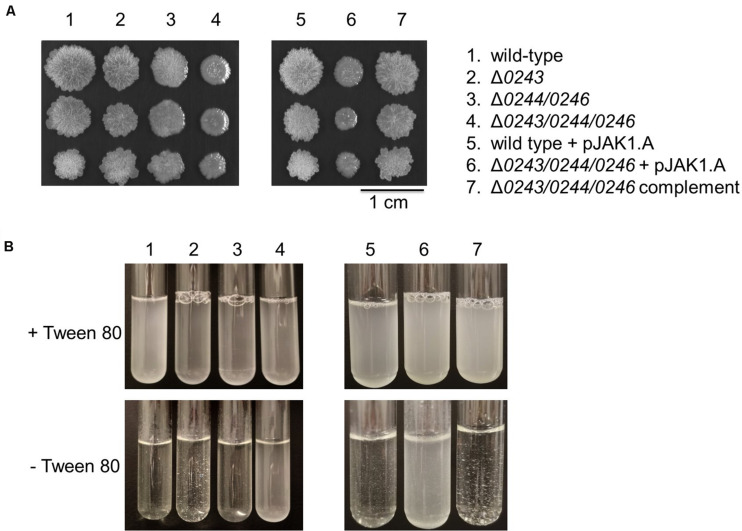
*M. smegmatis* strains were grown in 7H9 medium (liquid and solid). **(A)** Colony morphology after 6 days of growth on 7H9 agar at 37°C. 1 μL culture of OD_600_ 0.01, 0.001, and 0.0001 were spotted on the plate. Mutant Δ*0243/0244/0246* shows a smooth colony phenotype. **(B)** Liquid cultures of wild type cells, Δ*0243*, and Δ*0244/0246* displayed significant clumping in 7H9 medium without Tween 80 compared to Δ*0243/0244/0246*. This effect was reversed in the complemented strain. Tween 80 is a non-ionic emulsifier routinely added to mycobacterial liquid cultures in order to reduce cell aggregation (Lyon et al., 1963). All experiments were performed at least three times and one representative dataset is shown.

In addition, we cultured wild-type and knockout cells on solid agar to analyze differences in colony morphology. Interestingly, we found a profound phenotype of the mutant Δ*msmeg_0243/msmeg_0244/msmeg_0246*. Colonies were flat and less wrinkled than wild-type colonies (Figure 5A). The observed effect was restored to wild-type behavior in the complemented mutant strain.

Overall, in our experiments we did not observe any dependence of phenotypes on the supplied iron source (both in experiments with and without Tween 80) when investigating the three described mutants. However much more striking is the overall appearance of the biofilm and colonies when comparing the wild-type and knockout mutant Δ*msmeg_0243/msmeg_0244/msmeg_0246* independent of the supplied iron source. Interestingly, cultivation of cells in the absence of Tween 80 led to aggregation of wild-type cells, while cells of Δ*msmeg_0243/msmeg_0244/msmeg_0246* grew in suspension (Figure 5B). A recent study by DePas et al. shows that cell aggregation of non-tuberculous mycobacteria is a controlled process that is dictated by the relative availability of carbon and nitrogen sources. Differences in biofilm formation, clumping and colony appearance have been reported to be associated with changes in cellular lipid profiles in numerous studies. Along that line, we hypothesized that our mutants might possess differences in cell envelope lipid composition.

### Investigation of the Lipid Composition of *M. smegmatis Δmsmeg_0243/msmeg_ 0244/msmeg_0246, Δmsmeg_0244/msmeg_0246*, and *Δmsmeg_0243*

**FIGURE 6:**
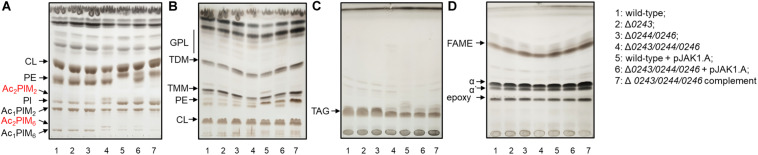
Lipid and mycolic acids analysis of 1: wild-type; 2: Δ*0243*; 3: Δ*0244/0246*; 4: Δ*0243/0244/0246*; 5: wild-type + pJAK1.A; 6: Δ*0243/0244/0246* + pJAK1.A; 7: Δ*0243/0244/0246* complemented grown in Sauton’s medium at 37°C for 3 days. The lipids were separated in **(A)** solvent I; **(B)** solvent II; **(C)** solvent III and mycolic acids were separated in **(D)** solvent IV. The lipids were visualized with 10% CuSO_4_ in 8% phosphoric acid solution and heating. Increase in amount of Ac_2_PIM_2_ and Ac_2_PIM_6_ was the only change that reproducibly correlated with the disruption of *MSMEG*_*0243/0244/0246* and was restored in the complemented strain. CL, cardiolipin; GPL, glycopeptidolipids; PE, phosphatidylethanolamine; PIM, phosphatidylinositol mannosides; PI, phosphatidylinositol; TMM, trehalose monomycolates; TDM, trehalose dimycolates; TAG, triacylglycerols; FAME, fatty acid methyl esters; α, α’ and epoxy refer to forms of mycolic acid methyl esters.

To that end, we isolated and analyzed total lipids and fatty/mycolic acids from wild-type and all studied strains grown on 7H11 or 7H9 agar at 37°C, as well as in the form of biofilms in Sauton’s medium at 37°C for 3 days and in M63 salts at 30°C for 5 days. In all of these conditions we observed different colony morphology or disturbed biofilm formation of Δ*msmeg_0243/msmeg_0244/msmeg_0246* mutant cells, while the other mutants grew similarly to the wild-type strain. Lipids were isolated using chloroform: methanol extraction followed by Folch wash (Folch et al., 1957), and subsequently analyzed by TLC (Figure 6 and Supplementary Figures S6,S7). Mycolic acids were released by saponification, derivatized to corresponding methyl esters and analyzed by TLC. This analysis did not show any reproducible differences in fatty and mycolic acids, mycolic acid derived molecules, glycopeptidolipids, triacylglycerols, phosphatidylethanolamine and cardiolipin, but in all tested growth conditions revealed higher amounts of acylated PIMs in Δ*msmeg_0243/msmeg_0244/msmeg_0246* mutant cells, especially of diacyl phosphatidylinositol dimannosides (Ac_2_PIM_2_) and diacyl phosphatidylinositol hexamannosides (Ac_2_PIM_6_).

## Discussion

In the described study, we investigate the potential role of the locus *msmeg_0243/msmeg_0244/msmeg_0246* from *M.sm* in heme/iron sensing. We report the expression, purification and *in vitro* heme binding properties of MSMEG_0243, an annotated periplasmic heme binding protein. Our presented follow-up experiments using the knockout mutant *M.sm*Δ*msmeg_0243* do not provide evidence of the observed *in vitro* heme-binding being linked to cellular heme uptake mechanisms, as it has been proposed for the homologous protein Rv0203 from *M.tb*. Hence, we hypothesized that heme binding of MSMEG_0243 might be linked to a sensing mechanism involving heme as chemical ligand (Girvan and Munro, 2013). There are several literature examples describing heme dependent sensing mechanisms of e.g., oxygen, nitrogen, carbon monoxide, radicals, redox state or light, as well as control mechanisms of heme synthesis itself or respiratory electron transport (Girvan and Munro, 2013). Sensing mechanisms of the described type are often involved in cellular stress homeostasis, virulence or cell wall homeostasis and important factors in host-cell interaction. As the homologous two component system PrrAB from *M.tb* has been implicated in early adaption upon macrophage infection (Ewann et al., 2004; Haydel et al., 2012; Mishra et al., 2017), a role in gas or radical sensing might be a potential function of the *msmeg_0243*/*msmeg_0244/msmeg_0246* locus. However, from our investigations we have no evidence that MSMEG_0244/MSMEG_0246 from *M.sm* is directly involved in a gas or radical sensing mechanism, as we did not observe differential growth response under anoxic, NO, CO, or H_2_O_2_ conditions in *M.sm*Δ*msmeg_0243*, Δ*msmeg_0244/msmeg_0246*, or Δ*msmeg_0243/msmeg_0244/msmeg_0246* mutants as compared to the wild-type strain. Interestingly, in our *in vitro* experiments we observed a differential binding affinity to MSMEG_0243 for heme depending on its oxidation state, and a characteristic absorbance spectrum for heme binding *via* an anionic ligand, as for example cysteine. Besides, Rv0203, the closest spectroscopic resemblance to our spectrum is found in the literature for anionic ligand binding penta-coordinated heme binding proteins. Interestingly, a similar atypically Q-band region is described for heme-binding GAF domains of sensor kinases from *Methanosarcina acetivorans*, where the heme cofactor is covalently bound to sGAF2 *via* a single cysteine residue (Fiege et al., 2018). The hypothesis that heme binding to MSMEG_0243 occurs *via* one of the cysteine residues that are found in MSMEG_0243 is intriguing, however, has been excluded for the homolog Rv0203 by Owens et al.(2012, p. 2), as both cysteine residues in the apo-protein (available crystal structure of Rv0203; PDB code 3MAY) are bridged by a disulfide bond. Nevertheless, we found several reports in the literature, in which a thiol-disulfide redox switch controls the affinity of a heme site (Ragsdale and Yi, 2011). These proteins are mainly involved in maintaining cellular or extracellular redox balance, ion-channel activity or heme degradation. Further studies in our laboratory are underway to finally clarify the heme binding motive and potential function as redox-switch of MSMEG_0243 from *M.sm*.

In our study, we do not find a direct link between heme/iron supplementation and growth characteristics of the described mutants, as we would expect from a potential role in heme/iron sensing. Yet, we observed a significant influence on biofilm formation, colony morphology and clumping phenotype in the mutant Δ*msmeg_0243/msmeg_0244/msmeg_0246.* Interestingly, we observe a pronounced phenotype only in the mutant Δ*msmeg_0243/msmeg_0244/msmeg_0246*, lacking both the two component system and the potential accessory protein MSMEG_0243. We can speculate that lack of *msmeg_0244*/*msmeg_0246* might be compensated by the close homologs on the *M.sm* genome, namely *msmeg_5662*/*msmeg_5663*. Sequence alignment reveals a sequence similarity of 72.5% for the transcriptional regulatory proteins MSMEG_5662 and MSMEG_0244 and for the sensor-type histidine kinases MSMEG_5663 and MSMEG_0246 of 57.4%. Moreover, the potential accessory protein MSMEG_0243 is missing in the genomic stretch of *msmeg_5662*/*msmeg_5663*, so in the absence of *msmeg_0244*/*msmeg_0246* it potentially interacts with *msmeg_5662*/*msmeg_5663.* MSMEG_0243 itself possesses several close homologs (e.g., MSMEG_5111, sequence similarity 70.0%) in *M.sm*, providing reason for the absence of observed phenotypes in the single mutant. Furthermore, it is worth mentioning that the double mutant Δ*msmeg_0244*/*msmeg_0246*, displays upregulated expression of *msmeg_0243* (Supplementary Figure S2B). Increased expression of the heme-binding protein is potentially compensating loss of the two-component system (or at least partially), hence leading to the lack in phenotypic effects observed for Δ*msmeg_0244*/*msmeg_0246*. Additionally, in the single mutant Δ*msmeg_0243* we observed a high upregulation of the prospective sensor-type kinase gene *msmeg_0246*, further pointing toward an interplay of the studied genes and indicating an attempt of the bacteria to re-optimize the system under the given conditions.

Our further investigations of cell envelope lipid profiles revealed that acylation of PIMs is significantly altered in the cells of Δ*msmeg_0243/msmeg_0244/msmeg_0246* mutant strain exhibiting changes in colony morphology or impaired biofilm formation. PIMs are one of the most abundant and bioactive glycolipid families in the mycobacterial cell wall. Acylated PIMs are found in the cell wall and cytoplasmic membrane of mycobacteria, they exhibit a wide spectrum of immune-regulatory effects and are known to play major roles in inducing granuloma and recruitment of natural killer cells (Gilleron et al., 2001; Court et al., 2011; Jackson, 2014). These molecules are essential structural components of the mycobacterial cell envelope, influencing permeability of the cell envelope to both hydrophilic and hydrophobic molecules, cell membrane integrity and additionally are involved in the regulation of cell septation and division (Guerin et al., 2010). Furthermore, PIMs production was reported to be affected by environmental factors known to impact replication rate and/or membrane fluidity, such as carbon/nitrogen sources and temperature (Taneja et al., 1979; Dhariwal et al., 2011). Just recently, PIMs biosynthesis has been linked to biofilm formation in mycobacteria, supporting our results (Li et al., 2019). Li et al. (2019) demonstrated that the transcription factor MpbR negatively regulates two transporter genes and modulates the accumulation of acylated PIMs in the cells of *M.tb*, which results in reduced lung burden and inflammation of infected mice. They also showed that the deletion of *mpbR* in *M.sm* affects the formation of biofilm. Furthermore, a knockout mutant of the universal stress protein Rv2623 in *M.tb* shows a smoother, less ruffled appearance of colonies as compared to wild-type cells, a phenomenon, which was also linked to a distinct PIMs profile with increased amount of mono- and diacylated forms of PIM_2_ and PIM_6_ in the mutant strain (Glass et al., 2017).

Affected biofilm formation was observed also in the case of knockout mutant *M.sm*Δ*mmpL11_S__M_* grown in the same conditions (Pacheco et al., 2013, p. 11). However, deletion of *mmpl1L*_SM_ was associated with lower levels of mycolic acid wax ester and long-chain triacylglycerols and did not lead to alteration of PIMs production further confirming that our observation is not evolving from any polar effect on *mmpL11_S__M_* gene. These considerations indicate that the two component system *msmeg_0244/msmeg_0246* and the potential accessory protein MSMEG_0243 might be involved in the regulation of genes encoding proteins carrying out important steps in PIMs acylation, a process which is required for cell envelope homeostasis. Biosynthesis of mycobacterial PIMs is crucial, as exemplified by essentiality of the mannosyltransferase PimA involved in the first mannosylation of PI in *M.sm*, as well as *M.tb* (Korduláková et al., 2002; Boldrin et al., 2014). Also disruption of PatA protein, catalyzing the acylation of phosphatidylinositol mono- and dimannosides, is lethal to *M.tb* H37Rv and results in severe growth defects in *M.sm* (Beier and Gross, 2006; Korduláková et al.,2003; Boldrin et al., 2014; Albesa-Jové et al., 2016). Enzymes involved in this pathway have been proposed to be promising drug targets and further insights into their regulation will provide a potential handle for future drug design.

Taken together from our presented data we can postulate a preliminary working model for the action of MSMEG_0244/MSMEG_0246 and MSMEG_0243 in *M.sm*. There is significant literature precedence that bacterial heme-binding proteins (McLean and Munro, 2017) are often involved in lipid homeostasis, synthesis and regulation (Girvan and Munro, 2013), and our data provides evidence that MSMEG_0243/MSMEG_0244/MSMEG_0246 work along the same line. MSMEG_0243 is possessing a N-terminal signal peptide indicating export *via* the Sec translocation pathway. Proteins are exported in an unfolded state, hence heme binding to MSMEG_0243 is most possibly occurring in the extracellular space. MSMEG_0243 might bind ferric heme and interact with the sensor kinase MSMEG_0246, which results in signal transduction to MSMEG_0244 and subsequent target gene regulation. Control of heme binding and as we postulate MSMEG_0246 interaction with MSMEG_0243 might occur by a mechanism that is involving reduction of ferric to ferrous heme. Upon dissociation of ferrous heme from MSMEG_0243, interaction with MSMEG_0246 is potentially altered, with downstream effects on enzymes involved in the synthesis of cell wall lipids. Future investigations might involve *in vitro* protein-protein interaction studies in the presence of ferric/ferrous heme molecules or pulldown-experiments using MSMEG_0243 as bait.

Our results underline the importance of two component systems and related proteins in mycobacteria. Our study points toward an interplay of a heme-binding accessory protein and a two component system in *M.sm*. Similar systems have been described in *Streptomyces, Bacillus subtilis*, or *Rhodococcus*, but to our knowledge have, to date, not been described in *Mycobacteria.* Disturbance in these genes results in the altered processing of essential components of the mycobacterial cell envelope, PIMs, in certain growth conditions, which are associated with severe defects in biofilm and colony formation.

## Data Availability Statement

All datasets presented in this study are included in the article/Supplementary Material.

## Author Contributions

JK and CJ-T design of the study and manuscript preparation. ML, SC, and XW: mutant construction and characterization. ML and SC: cloning, protein purification and characterization. HG: lipid analysis. All authors read and approved the final version of the manuscript.

## Conflict of Interest

The authors declare that the research was conducted in the absence of any commercial or financial relationships that could be construed as a potential conflict of interest.

## Abbreviations

ADC: albumin-dextrose-catalase
CL: cardiolipin
DMSO: dimethyl sulfoxid
FAME: fatty acid methyl esters
IPTG: isopropyl- β-D -thiogalactopyranoside
LB: lysogeny broth
MIC: minimal inhibitory concentration
*M.sm*: *Mycobacterium smegmatis*
*M.tb*: *Mycobacterium tuberculosis;*
OADC: oleic acid-albumin-dextrose-catalase
PCR: polymerase chain reaction
PE: phosphatidylethanolamine
PI: phosphatidylinositol
PIM: phosphatidylinositol mannosides
RT-PCR: reverse transcribe PCR
SDS-PAGE: sodium dodecyl sulfate polyacrylamide gel electrophoresis
SDT: sodium dithionite
SM: Sauton’s medium
TAG: triacylglycerols
TLC: thin layer chromatography
TB: tuberculosis
TDM: trehalose dimycolates
TMM: trehalose monomycolates
X-GAL: 5-brom-4-chlor-3-indoxyl- β-D -galactopyranosid.
