# Supplementary material for: The Two-Component Locus MSMEG_0244/0246 Together With MSMEG_0243 Affects Biofilm Assembly in M. smegmatis Correlating With Changes in Phosphatidylinositol Mannosides Acylation
Source: Front Microbiol. 2020 Sep 11;11:570606. doi: 10.3389/fmicb.2020.570606 (PMC7516205; doi:10.3389/fmicb.2020.570606)
Supplement: Supplementary file 1 [file Data_Sheet_1.pdf]

Supplementary Material

The two-component locus MSMEG_0244/0246 together with MSMEG_0243 affects biofilm assembly in *M. smegmatis* correlating with changes in phosphatidylinositol mannosides acylation

Miaomaio Li[1], Henrich Gašparovič[2], Xing Weng[1], Si Chen[1], Jana Korduláková[2]* & Claudia Jessen-Trefzer[1]*

[1]Department of Pharmaceutical Biology and Biotechnology, University of Freiburg, Stefan-Meier-Str. 19, 79104 Freiburg, Germany.

[2]Department of Biochemistry, Faculty of Natural Sciences, Comenius University in Bratislava, Mlynská dolina, Ilkovičova 6, 842 15 Bratislava, Slovakia.

*** Correspondence:**
Jana Korduláková and Claudia Jessen-Trefzer, E-mail: jana.kordulakova@uniba.sk and claudia.jessen-trefzer@pharmazie.uni-freiburg.de

1 Supplementary Figures and Tables

1.1 Supplementary Tables

Table S 1: Strains, plasmids and primers used in this study.

**Strains**	**Origin**
M. smegmatis mc^2155	Gift from W. Jacobs lab (Albert Einstein College of Medicine, USA)
M. smegmatis mc^2155 Δmsmeg_0243	This study
M. smegmatis mc^2155 Δmsmeg_0244/ msmeg_0246	This study
M. smegmatis mc^2155 Δmsmeg_0243/msmeg_0244/msmeg_0246	This study
M. smegmatis mc^2155 + pJAK1.A	This study
M. smegmatis mc^2155 Δmsmeg_0243/msmeg_0244/msmeg_0246 + pJAK1.A	This study
M. smegmatis mc^2155 Δmsmeg_0243/msmeg_0244/msmeg_0246 + pJAK1.A::msmeg_0243/44/46	This study
**Plasmids**	
pET-22b (+)	Novagen (Merk)
pJAK1.A	Addgene, #29460

pJAK1.A::msmeg_0243/0244/0246	This study
P1NIL	Addgene, #20187
pGOAL19	Addgene, #20190
P1NIL-Δ0243	This study
P1NIL-Δ0243-RM	This study
P1NIL-Δ0244/0246	This study
P1NIL-Δ0244/0246-RM	This study
P1NIL-Δ0243/0244/0246	This study
P1NIL-Δ0243/0244/0246-RM	This study
**Primers**	
MSMEG_0243_Strep-tag_FW	CCCATGGATCCGAACCCGCCCAACTGCAC (*Nco*I)
MSMEG_0243_Strep-tag_REV	GGCCGCAAGCTTTCA CTT TTC GAA CTG CGG GTGGCTCCACTCGAGTGCGGCCGCCGGCAATTC AGGTGCCGG (*Hind*III)
SigA control_FW	CGACTACACCAAGGGCTACAAGTTC
SigA control_REV	CGATCGTCTGGTCCAGCGAGATG
Check-intergenic region 1_FW	GAGGTGAAGCCCAAGGTTG
Check-intergenic region 1_REV	ATCGTGCGACTCCGTCGA
Check-intergenic region 2_FW	GCGTCGACCTGACCAAG

Check-intergenic region 2_REV	ACCACCGACGTCAGGATC
Δmsmeg_0243_upstream_FW	GCTTTAATTAATAGCGTCTGCACGCCCTGTAG (*Pac*I)
Δmsmeg_0243_upstream_REV	GCTGGTACCACTAGTCACTCGCCGCGCATTCTG (*Kpn*I)
Δmsmeg_0243_downstream_FW	GCTACTAGTTAGTCCGCGTGCGGGTG (*Spe*I)
Δmsmeg_0243_downstream_REV	GCTAAGCTTACCGGTGATCGGATCGGTG (*Hind*III)
Δmsmeg_0244/0246_upstream_FW	GCTTTAATTAAAACTGGACGTTGGTGGTCTCG (*Pac*I)
Δmsmeg_0244/0246_upstream_REV	GCTGGTACCACTAGTCACGCGGACTACGGCAATTC (*Kpn*I)
Δmsmeg_0244/0246_downstream_FW	GCTACTAGTACGGATCAGTTGGCCTGAG (*Spe*I)
Δmsmeg_0244/0246_downstream_REV	GCTAAGCTTTGCACGGCGATGATGAGAC (*Hind*III)
Δmsmeg_0243/0244/0246_upstream_FW	GCTTTAATTAATAGCGTCTGCACGCCCTGTAG (*Pac*I)
Δmsmeg_0243/0244/0246_upstream_REV	GCTGGTACCACTAGTCACTCGCCGCGCATTCTG (*Kpn*I)
Δmsmeg_0243/0244/0246_downstream_FW	GCTACTAGTACGGATCAGTTGGCCTGAG (*Spe*I)
Δmsmeg_0243/0244/0246_downstream_REV	GCTAAGCTTTGCACGGCGATGATGAGAC (*Hind*III)
Δmsmeg_0243_check ko_FW (MS181)	AGACCAACCAGGCTCTGAC

Δmsmeg_0243_check ko_REV (MS182)	TCCGACTGTCATGGTGTC
Δmsmeg_0244/0246_check ko_FW(MS183)	GAGCTGCAATTTCGATGC
Δmsmeg_0244/0246_check ko_REV (MS184)	GACGTATGCGAGCGACTAG
Δmsmeg_0243/0244/0246_check ko_FW (MS185)	TCGACGACTGGTTGAGAG
Δmsmeg_0243/0244/0246_check ko_REV (MS186)	GGGCTACCGTTTGTGTTC
comp_0243/0244/0246_FW	GGGGACAAGTTTGTACAAAAAAGCAGGCTTCGA AGGAGATAGAACCATGGTG AAGGCCGCCATCA
comp_0243/0244/0246_REV	GGGGACCACTTTGTACAAGAAAGCTGGGTCTCA GGCCAACTGATCC
msmeg_0241_expression_FW	AACTGGACGTTGGTGGTCTCG
msmeg_0241_expression_REV	GATGTTCGACCAGTGGAACTG
msmeg_0243_expression_FW	CCCATGGATCCGGCCCCGAAC
msmeg_0243_expression_REV	CCCTCGAGCGGCAATTCAGGTGCCGG
msmeg_0244_expression_FW	AATCGCACGGTCCTCATG
msmeg_0244_expression_REV	TCCGACTGTCATGGTGTC
msmeg_0246_expression_FW	ATAGCAACGATGCGGCACAAC
msmeg_0246_expression_REV	CGGAATCCGGTTGGCGCT

1.2 Supplementary Figures

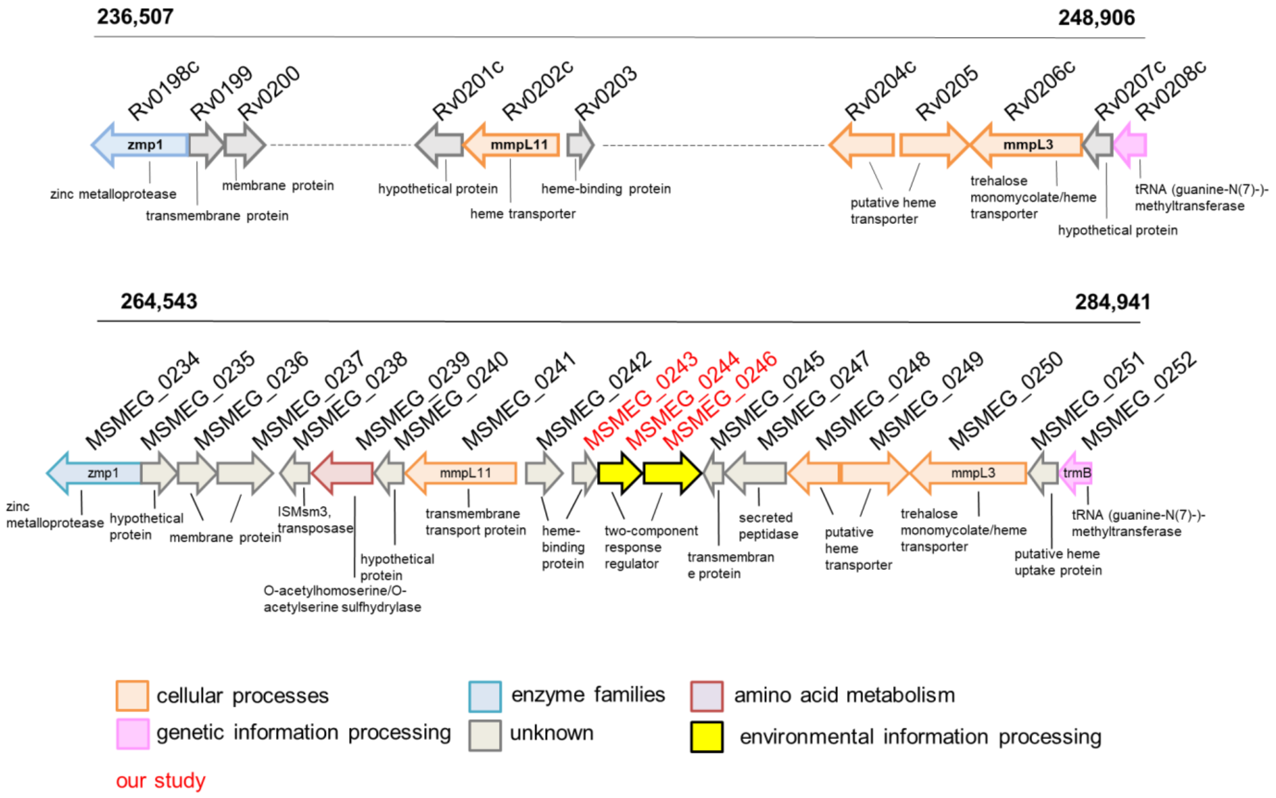

Figure S 1: Comparing the genomic locus surrounding *mmpL11* and *mmpL3* in *M. tuberculosis* and *M. smegmatis*.

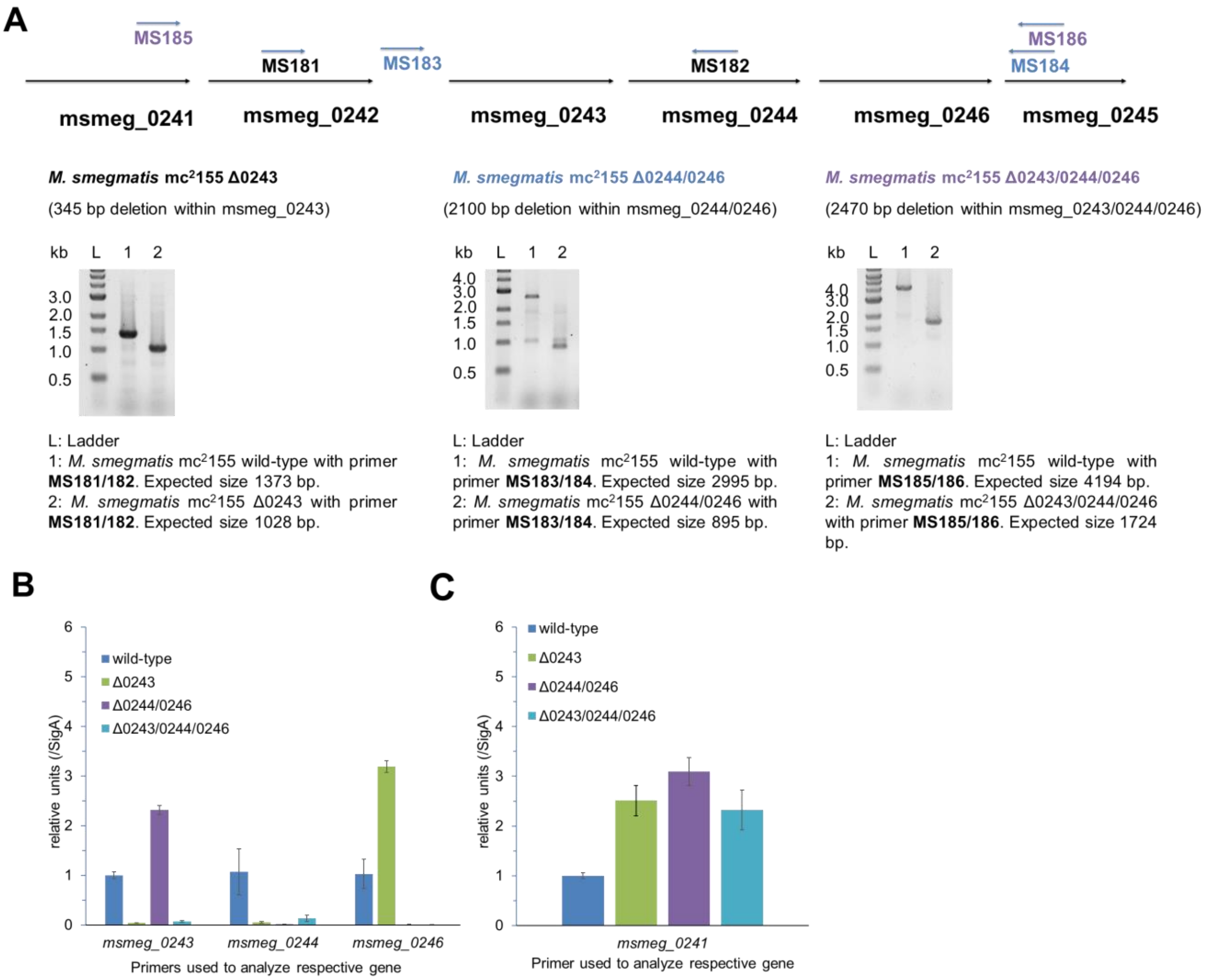

Figure S 2: Validation of knockout mutants by PCR analysis using the primer pairs as indicated in Table S1 and analysis of the KO mutants by RT-PCR. A) Genomic DNA isolated of wild-type cells and the respective knockout mutant was amplified by PCR using primers amplifying a large region around the expected gene knockout. The PCR products were confirmed by Sanger sequencing. B) Expression of the genes *msmeg_0243*, *msmeg_0244*, *msmeg_0246* and C) *msmeg_0241* (*mmpL11*) was analyzed by RT-PCR using primer pairs as indicated in Table S1. Wild-type and knockout mutants are compared in their expression level of the respective genes. The experiment was performed in triplicates. Expression levels were normalized to SigA. Mean values indicated; error bars indicate ± SEM.

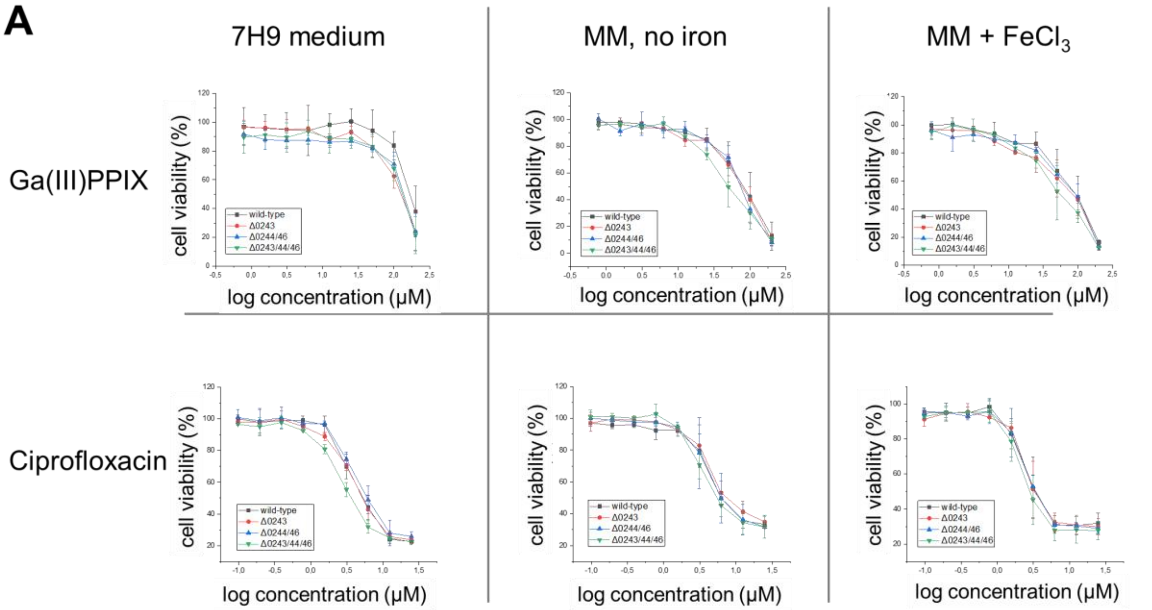

IC $_{50}$ (µM)	7H9 medium		MM, no iron		MM with $FeCl_3$	
	Ga(III)PPIX	Ciprofloxacin	Ga(III)PPIX	Ciprofloxacin	Ga(III)PPIX	Ciprofloxacin
wild-type	115.3 ± 17.9	9.0 ± 0.9	73.2 ± 24.0	10.8 ± 0.9	65.9 ± 9.5	5.3 ± 1.6
Δ0243	97.9 ± 15.4	7.1 ± 1.1	70.4 ± 7.6	12.2 ± 1.6	45.4 ± 4.8	6.7 ± 1.8
Δ0244/0246	121.8 ± 26.5	9.8 ± 2.1	64.5 ± 8.3	10.6 ± 2.4	63.5 ± 4.8	3.8 ± 0.5
Δ0243/0244/0246	111.7 ± 10.5	5.3 ± 0.3	46.2 ± 5.2	8.2 ± 1.2	48.5 ± 4.8	5.0 ± 1.6

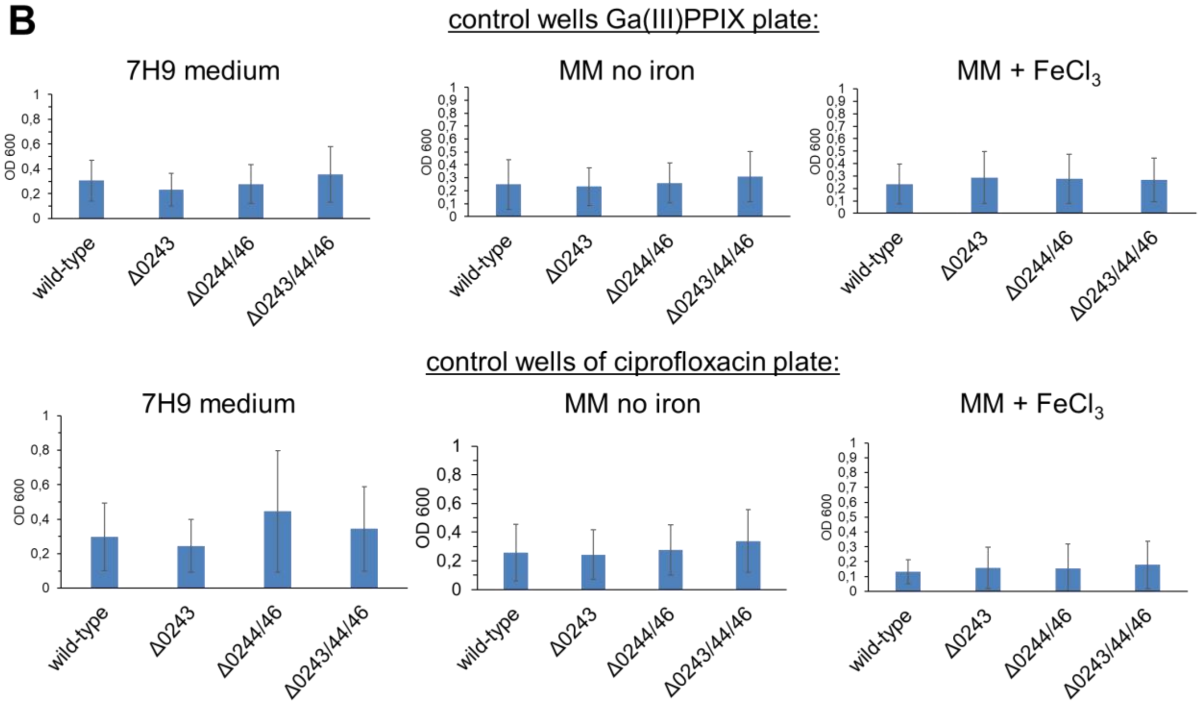

Figure S 3: Viability assay with Ciprofloxacin and Ga(III)PPIX in different growth medium containing high (7H9 medium, 150 µM) or low concentrations (modified Sauton's medium, 10 µM)

of iron(III). A) *M. smegmatis* mc² 155 wild-type and knockout mutants were incubated with the respective compound in 96-well plate format. Cell viability after 24 hours was determined using the resazurin reduction assay (fluorescence readout at 560 nm excitation / 590 nm emission). Cell viability (in %) is plotted *vs* log concentration (µM). Table summarizes IC_{50} values. Data represent means of triplicates ±SEM. MM: Minimal Medium, PPIX: protophorphyrin IX. Black line: wild-type, red line: Δ0243, blue line: Δ0244/0246 and green line: Δ0243/0244/0246. B) Control cell growth measurements in different medium applied during cell viability assays (no drug added in control wells). Growth of *M. sm.* wide-type and knockout mutants in the indicated medium was monitored in 96-well plate format with a starting OD600 of 0.05. OD was re-determined after 24 hours. Data represent means of triplicates ±SEM. MM: Minimal medium = modified Sauton‘s medium.

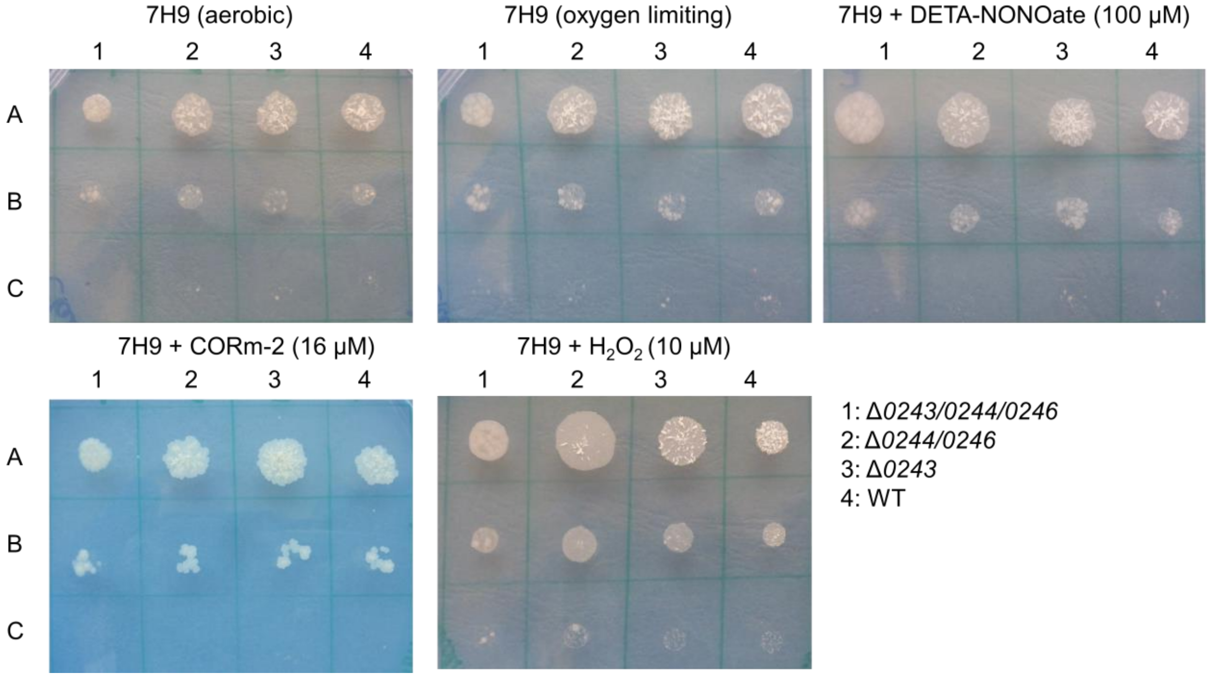

Figure S 4: Top-down view of spotting assay of *M. smegmatis* mc^{2}155 wild-type and knockout mutants assaying various growth conditions. Dilutions of cells (OD: A:10^{-2}, B:10^{-3} and C:10^{-4}) were spotted (1 µL) on 7H9 (detergent free) agar with either no supplement or DETA-NONOat (NO donor), CORm-2 (CO donor) or H_2O_2. Plates were incubated under aerobic or oxygen limiting conditions at 37°C for 2 days. The assay was repeated three times yielding similar results. One representative dataset is shown.

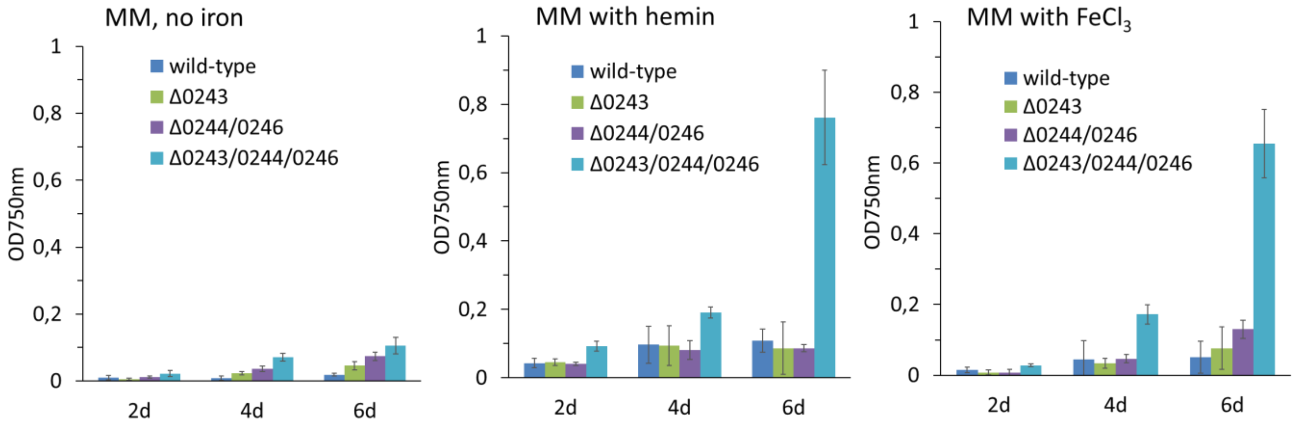

Figure S 5: Control cell growth measurements in different medium applied during biofilm assessment. Planktonic grown bacteria at the bottom of the plate were re-suspended in 7H9 containing Tween 80 and quantified by OD750 measurements. The measurements were performed at day 2, day 4 and day 6. Data represent means of triplicates ±SEM. MM: Minimal medium=modified Sauton's medium; d: day; dark blue: wild-type; green: Δ0243; violet: Δ0244/0246 and light blue: Δ0243/0244/0246.

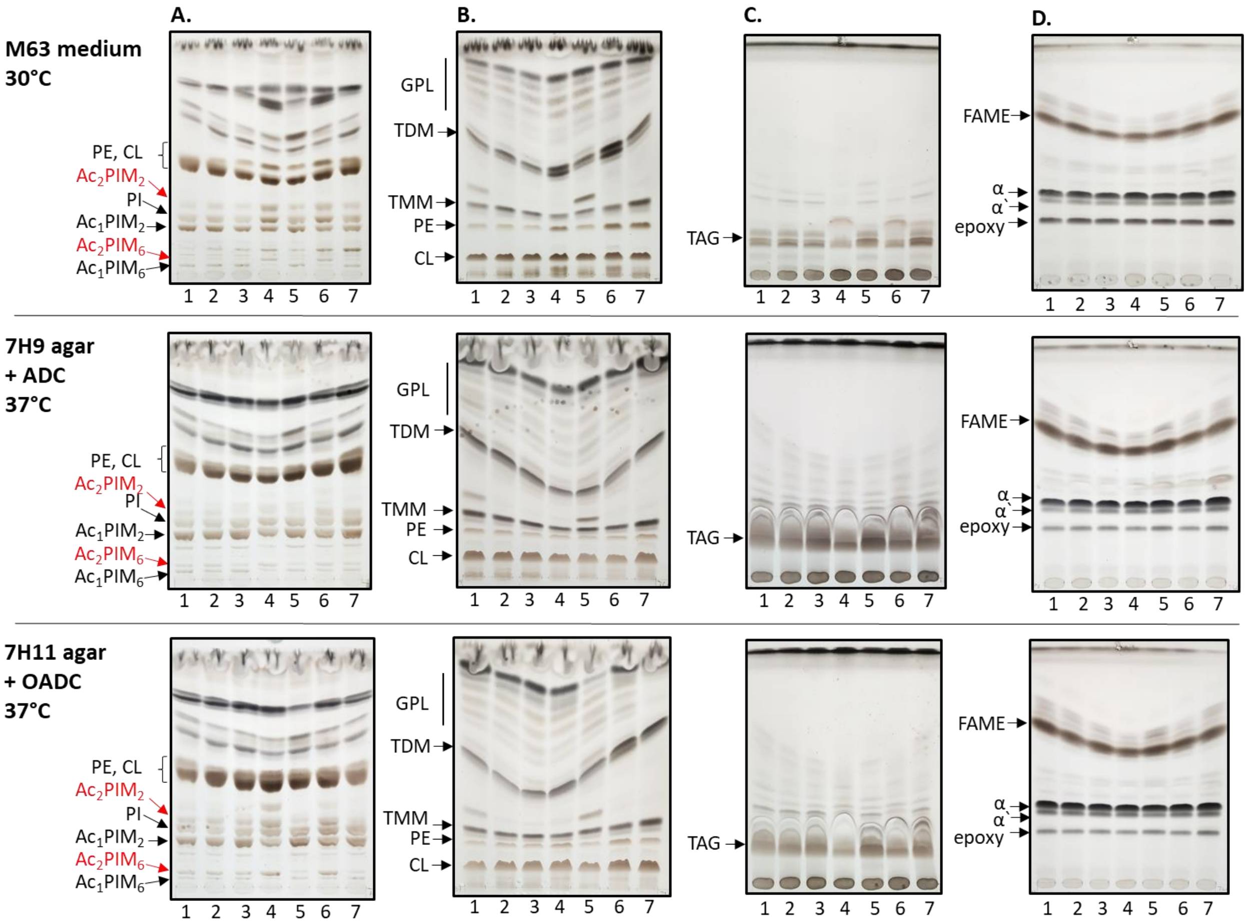

Figure S 6: Lipid and mycolic acids analysis of 1: wild-type; 2: Δ0243; 3: Δ0244/0246; 4: Δ0243/0244/0246; 5: wild-type + pJAK1.A; 6: Δ0243/0244/0246 + pJAK1.A; 7: Δ0243/0244/0246 complemented grown in different conditions. The lipids were separated in A: solvent I; B: solvent II; C: solvent III and mycolic acids were separated in C: solvent IV. The lipids were visualized with 10% $CuSO_4$ in 8% phosphoric acid solution and heating. CL: cardiolipin; GPL: glycopeptidolipids; PE: phosphatidylethanolamine; PIM: phosphatidylinositol mannosides; PI: phosphatidylinositol; TMM: trehalose monomycolates; TDM: trehalose dimycolates; TAG: triacylglycerols; FAME: fatty acid methyl esters; a, a' and epoxy refer to forms of mycolic acid methyl esters.

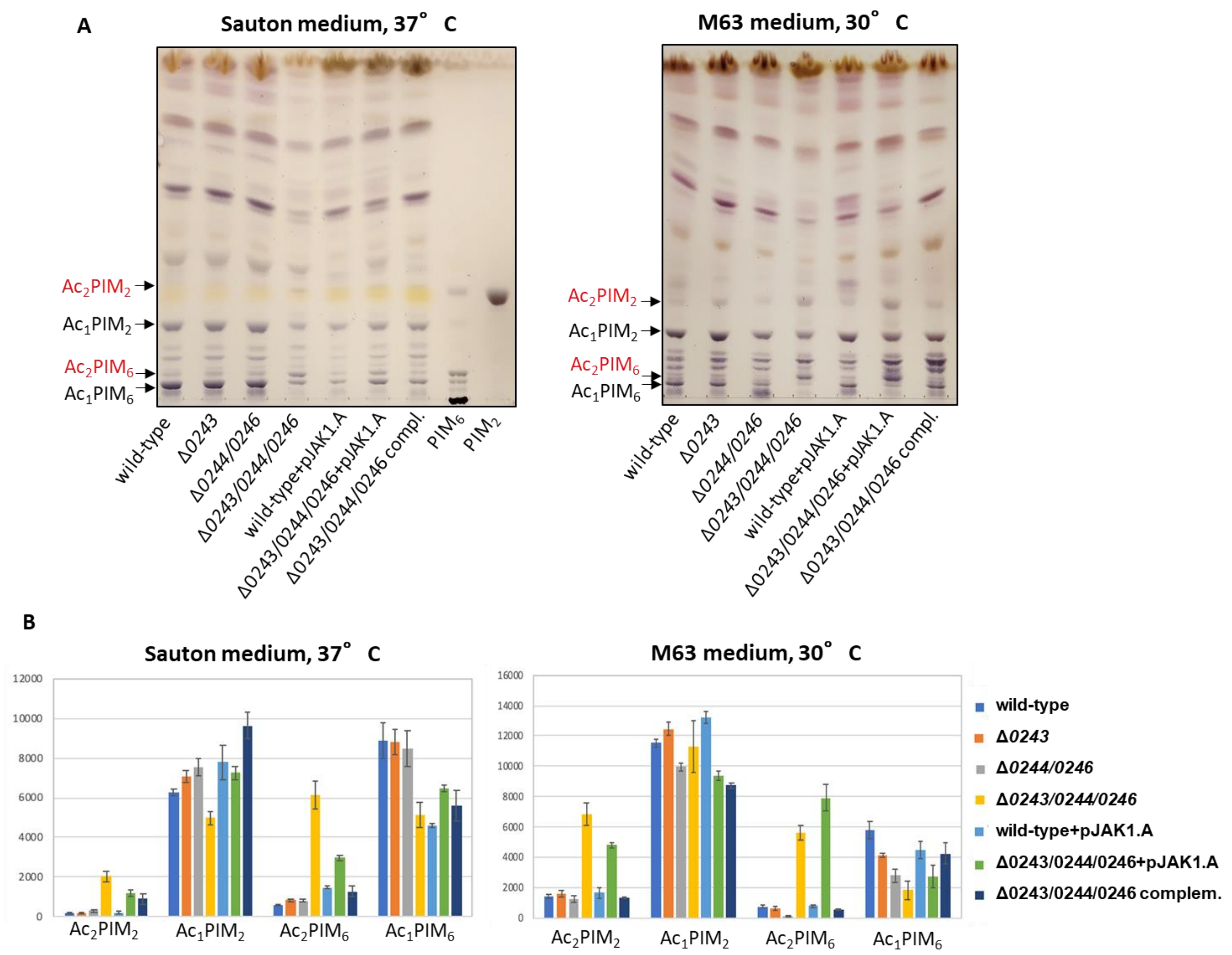

Figure S 7: Identification and quantification of different forms of phosphatidylinositol mannosides in lipid samples of studied strains grown as biofilms in Sauton at 37°C or M63 at 30°C. A) TLC analysis of lipids separated in solvent I and visualized with 0.5% (w/v) α-naphtol in 5% (v/v) sulphuric acid in ethanol and heating. PIM_2: The following reagent was obtained through BEI Resources, NIAID, NIH: *Mycobacterium tuberculosis*, Strain H37Rv, Purified Phosphatidylinositol Mannosides 1 & 2 (PIM1,2), NR-14846; PIM_6: The following reagent was obtained through BEI Resources, NIAID, NIH: *Mycobacterium tuberculosis*, Strain H37Rv, Purified Phosphatidylinositol Mannoside 6 (PIM6), NR-14847. B) Bar graph represents quantification of each PIM population using ImageJ software. The same samples were analyzed by three independent TLC analysis, lipids were separated and detected as described above and the intensity of each band was quantified using ImageJ software. Error bars represents +/- SEM. PIM: phosphatidylinositol mannosides.
